# A GCSFR/CSF3R zebrafish mutant models the persistent basal neutrophil deficiency of severe congenital neutropenia

**DOI:** 10.1038/srep44455

**Published:** 2017-03-10

**Authors:** Vahid Pazhakh, Sharon Clark, M. Cristina Keightley, Graham J. Lieschke

**Affiliations:** 1 Australian Regenerative Medicine Institute, Monash University, Clayton, Victoria 3800, Australia

## Abstract

Granulocyte colony-stimulating factor (GCSF) and its receptor (GCSFR), also known as CSF3 and CSF3R, are required to maintain normal neutrophil numbers during basal and emergency granulopoiesis in humans, mice and zebrafish. Previous studies identified two zebrafish CSF3 ligands and a single CSF3 receptor. Transient antisense morpholino oligonucleotide knockdown of both these ligands and receptor reduces neutrophil numbers in zebrafish embryos, a technique widely used to evaluate neutrophil contributions to models of infection, inflammation and regeneration. We created an allelic series of zebrafish *csf3r* mutants by CRISPR/Cas9 mutagenesis targeting *csf3r* exon 2. Biallelic *csf3r* mutant embryos are viable and have normal early survival, despite a substantial reduction of their neutrophil population size, and normal macrophage abundance. Heterozygotes have a haploinsufficiency phenotype with an intermediate reduction in neutrophil numbers. *csf3r* mutants are viable as adults, with a 50% reduction in tissue neutrophil density and a substantial reduction in the number of myeloid cells in the kidney marrow. These *csf3r* mutants are a new animal model of human CSF3R-dependent congenital neutropenia. Furthermore, they will be valuable for studying the impact of neutrophil loss in the context of other zebrafish disease models by providing a genetically stable, persistent, reproducible neutrophil deficiency state throughout life.

Granulocyte colony-stimulating factor (GCSF), also known as Colony-stimulating Factor 3 (CSF3), is a key regulator of neutrophil production and a wide range of neutrophil functions such as migration, antimicrobial activities and neutrophil survival^1^. These primary roles of GCSF in neutrophil cell biology are evolutionarily conserved between mammals such as humans and mice as well as fish including zebrafish^2^. GCSF signalling is initiated from the GCSF receptor (GCSFR), a class 1 cytokine receptor, and engages intracellular mediators, commonly the JAK/STAT/SOCS pathway.

The absolute requirement for GCSF signalling in granulopoiesis was first demonstrated by GCSF and GCSFR deficient mice, which have neutrophil and myeloid progenitor cell deficiencies, and exhibit vulnerability to infective challenges^3^,^4^. A rare form of human congenital neutropenia is due to biallelic *GCSFR* mutations^5^,^6^. Somatic *GCSFR* mutations are frequently acquired in long-standing GCSF-treated congenital neutropenia patients, and are associated with progression to acute myeloid leukaemia^7^.

Zebrafish granulopoiesis, at both primitive and definitive stages, is regulated through many cellular and molecular mechanisms that are largely conserved with mammalian granulopoiesis^8^,^9^. Hence, zebrafish models of myeloid development and neutrophil function have been exploited to gain new insights into the genetic and molecular regulation of neutrophil development, and the role of neutrophils in inflammatory and infective disease models. Specifically, GCSF/GCSFR signalling is conserved in zebrafish^2^,^10^ with two zebrafish GCSF/CSF3 ligands encoded by genes on chromosomes 12 (designated *csf3a*, ZFIN ID: ZDB-GENE-091229-1) and 19 (designated *csf3b*, ZFIN ID: ZDB-GENE-141212-221), which signal through a single receptor encoded by a gene located on chromosome 16 (*csf3r*, ZFIN ID: ZDB-GENE-080104-4). The ligands display different spatiotemporal expression patterns suggesting individualized function^10^. Indeed, a specific role for *csf3b* has been demonstrated in the later phase of neutrophil migration during the response to tissue injury^11^.

The requirement for both Csf3 ligands and Csf3r in zebrafish granulopoiesis has been demonstrated by transient loss-of-function studies employing antisense morpholino oligonucleotide knockdown strategies, which result in transient neutrophil depletion in zebrafish embryos^2^,^10^. The sufficiency of Csf3 signalling in adult zebrafish granulopoiesis is demonstrated by activity of the Csf3 ligands to support the *in vitro* development of myeloid-cell containing haemopoietic colonies^10^.

Here we describe the generation and characterisation of zebrafish *gcsfr* mutants using targeted CRISPR/Cas9 mutagenesis. Zebrafish *csf3r* mutants have a profound and stable neutrophil deficiency as embryos. The impairment of granulopoiesis persists into adulthood, manifesting as marked reduction of neutrophil abundance in kidney haematopoietic marrow and peripheral tissues. These studies confirm the primary role of Csf3/Csf3r signalling in granulopoiesis in zebrafish, and provide a new tool for assessing the contribution of neutrophils in embryonic and adult zebrafish disease models. Unlike transient knockdown approaches, which require potentially confounding experimental manipulations to induce neutrophil depletion, these *csf3r* mutants intrinsically provide a stable, basal neutrophil deficiency state *in vivo*.

## Results

### CRISPR/Cas9 mutagenesis of zebrafish *csf3r*

**Figure 1:**
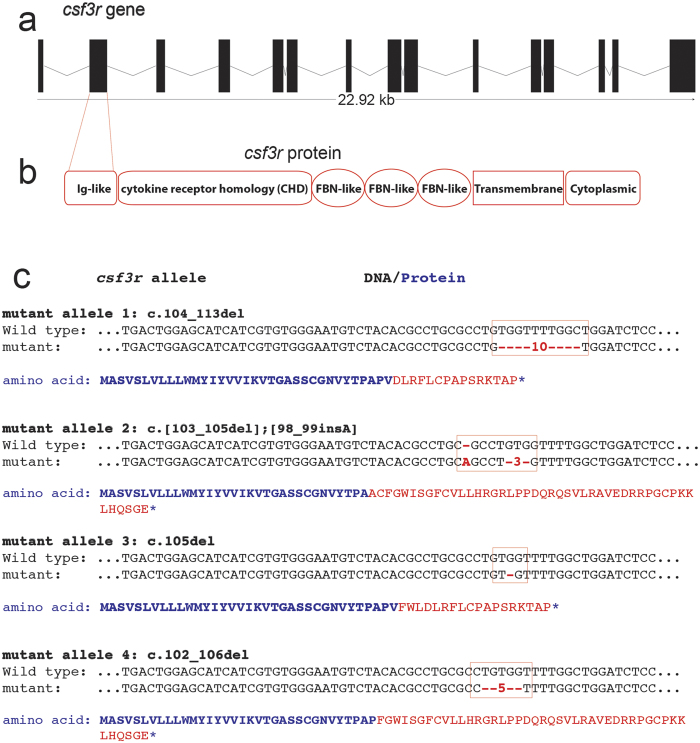
CRISPR/Cas9-induced mutant zebrafish *csf3r* alleles. (**a**) Intron/exon structure of zebrafish *csf3r* locus. (**b**) Domain structure of zebrafish Csf3r protein. Ig = immunoglobulin, FBN = fibronectin. (**c**) Four CRISPR/Cas9-induced *csf3r* nonsense mutations identified in adult F1 DNA (designated alleles 1–4 for this report) aligned to WT sequence. The corresponding predicted truncated amino acid sequences are shown: blue = native Csf3r sequence, red = predicted non-native sequence downstream of the mutation site, *premature stop.

Three sgRNAs (C1, C2 and C3) designed to target exon 2 in the *csf3r* gene were injected (Fig. 1a,b, Supplementary Fig. S1). Only C3 sgRNA resulted in mutagenesis at the expected target site in F0 sgRNA-injected embryos. On-target mutagenesis in these F0 embryos was confirmed by sequencing the predicted target site in a cohort of embryos, which revealed corrupted sequence traces commencing in the vicinity of the sgRNA target sequence for the C3 sgRNA (Supplementary Fig. S2), but not for sgRNAs C1 or C2.

Adult F0 fish from sgRNA C3 injections were incrossed to enable immediate observation of the predicted phenotype of reduced neutrophil numbers in F1 embryos, although genetic complexity was anticipated due to a multiplicity of CRISPR/Cas9-induced mutations. Germline transmission of mutant alleles was confirmed by genotyping F1 embryos and observing duplex sequencing traces at the predicted target sites (Fig. 1). Genotyping of individual adult F1 fish revealed multiple *csf3r* alleles, four of which were selected for propagation and future study (designated alleles 1–4 for this report, Fig. 1c). These four *csf3r* mutant alleles in exon 2 were all predicted to result in premature stop codons leading to Csf3r proteins truncated within the extracellular immunoglobulin (Ig)-like domain (Fig. 1b,c) and are currently undergoing genetic segregation by outcrossing. The studies presented here are a combination of analyses of F1 embryos and adults arising from F0 incrosses, and of F2 embryos and adults arising from F1 incrosses, and hence include biallelic mutants of several compound heterozygous and homozygous genotypes that randomly resulted from mating the first-available cohort of genotyped fish. Specific *csf3r*^*−/−*^ allelotypes contributing to each experiment are specified throughout.

### *csf3r* mutant zebrafish embryos have a selective, persistent neutrophil deficiency

To facilitate recognition of a neutrophil-depletion phenotype, mutagenesis was performed on a *Tg(mpx:EGFP)* background where neutrophils are marked by EGFP fluorescence. Even as F0 embryos, populations of C3 sgRNA-injected F0 embryos had significantly fewer *Tg(mpx:EGFP)-*labelled neutrophils compared to uninjected controls and embryos injected with either C1 and C2 sgRNAs (Supplementary Fig. S2). The occurrence of a neutrophil-depletion phenotype only in C3-injected F0 embryos also correlated with C3 being the only sgRNA for which targeting of the *csfr3r* locus was successfully demonstrated by Sanger sequencing. Hence, despite their complex mosaic genotype and heterogeneous mix of numerous *csf3r* allelic variants (evidenced in individual F0 embryo sequences, Supplementary Fig. S2), the expected *csf3r*-null phenotype of neutrophil deficiency was clearly discernable in F0 embryos when *csfr3r* targeting occurred.

**Figure 2:**
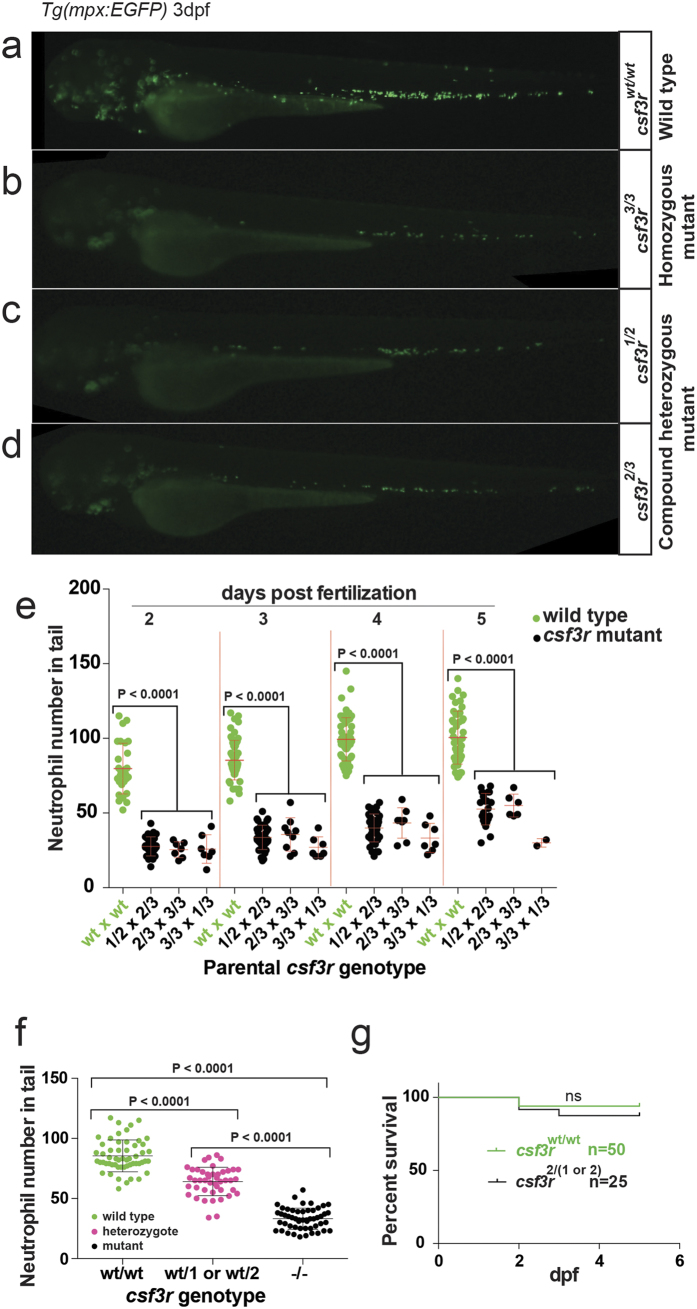
Neutrophil deficiency in *csf3r* null F2 embryos. (**a**–**d**) Fluorescence micrographs of 3 dpf *Tg(mpx:EGFP)* embryos showing that several representative *csf3r* mutant null allelotypes (**b**–**d**) have substantially reduced numbers of fluorescent neutrophils compared to WT (**a**). Panel **b** is a representative homozygous *csf3r*^*3/3*^ null embryo, and panels **c-d** show representative compound heterozygous null embryos of the two allelotypes shown. (**e**) Quantification over 2–5 days post-fertilisation (dpf) of tail region neutrophil numbers in embryos of control (WT) matings and incrosses of 3 different pairs of *csf3r* null parents of the *csf3r* allelotypes shown. Data are mean ± SD. Within-day comparisons across genotypes are analysed by unpaired two-tailed t-tests, pooling data from the mutant compound heterozygous allelotypes shown, which are not significantly different to each other. These parental allelotype combinations were randomly selected, being those pairs that laid of those that were set up. They are not intended to be comprehensive, but they do demonstrate a consistent non-complementing neutrophil-depletion phenotype encompassing five embryonic allelotypes (1/2, 1/3, 2/2, 2/3 and 3/3). (**f**) Embryos carrying single *csf3r* null alleles have an intermediate neutrophil deficiency. A mix of heterozygous *csf3r*^*wt/(1or2)*^ embryos in a 1:1 ratio were generated by outcrossing a parent of genotype *csf3r*^*1/2*^ to WT. Their neutrophil numbers at 3 dpf are compared with the non-contemporaneous *csf3r*^*WT/WT*^ and pooled *csf3r*^*−/−*^ mutant 3 dpf groups of panel (**e**). The mutant data were pooled as none of the mutant allelotype groups is significantly different to any other. p-values are from one-way ANOVA with Tukey’s multiple comparisons test. p < 0.0001. (**g**) *csf3r* null embryos have equivalent survival to WT embryos up to 5 days post-fertilisation (dpf). Kaplan-Meier plots compared by Wilcoxon rank sum test.

Adult F1 fish from F0 incrosses were genotyped and 6 fish carrying two *csf3r* null alleles were identified representing one homozygous and 3 compound heterozygous *csf3r* null genotypes (specific allelotypes: *csf3r*^*3/3*^, *csf3r*^*1/2*^, *csf3r*^*2/3*^, and *csf3r*^*1/3*^). Incrosses of these fish generated null mutant F2 embryos of multiple compound *csf3r* mutant allelotypes, all of which showed obvious neutrophil deficiency upon inspection, with neutrophil population sizes being 30%, 36%, 38% and 45% of normal at 2, 3, 4 and 5 dpf, respectively (p < 0.05) (Fig. 2a–e). The concordance of the phenotype across the multiple compound heterozygous and homozygous allelotypes represented in these crosses demonstrates non-complementation of the various alleles. It also provides genetic evidence for the specificity of the neutrophil depletion phenotype to on-target mutagenesis at the GCSFR locus and demonstrates the robustness of this genotype/phenotype association.

To test if there was a gene dosage-dependent neutrophil phenotype, a 1:1 mixture of heterozygous *csf3r*^*WT/1*^ or *csf3r*^*WT/2*^ F2 embryos was generated from an outcross of compound heterozygous fish A (*csf3r*^*1/2*^), and tail neut-ro-phil population sizes at 3 dpf compared with the pooled historical control data from 3 dpf *csf3r*^*WT/WT*^ embryos and a heterogenous mix of *csfr3r*^*−/−*^ allelotypes of Fig. 2e. WT (*csf3r*^*WT/WT*^), heterozygous carriers (*csf3r*^*WT/(1or2)*^) and *csf3r*^*−/−*^ nulls had 85.4 ± 13.3, 64.1 ± 11.8 and 33.7 ± 8.2 neutrophils per tail region respectively, consistent with an intermediate haploinsufficiency phenotype (Fig. 2f). Also of note, the range of tail neutrophil numbers did not overlap between WT and mutant null embryos (WT:58–117 n = 55; null:18–57 neutrophils, n = 36). Furthermore, the upper 40% of WT and the lower 56% of *csf3r*^*1/2*^ mutant neutrophil counts fell outside the heterozygote range, these groups consistently reflecting their csf3r gene dosage.

Survival of *csf3r* mutant neutrophil-depleted embryos over this period was normal (Fig. 2g), indicating that neutrophil deficiency did not affect early embryo viability.

**Figure 3:**
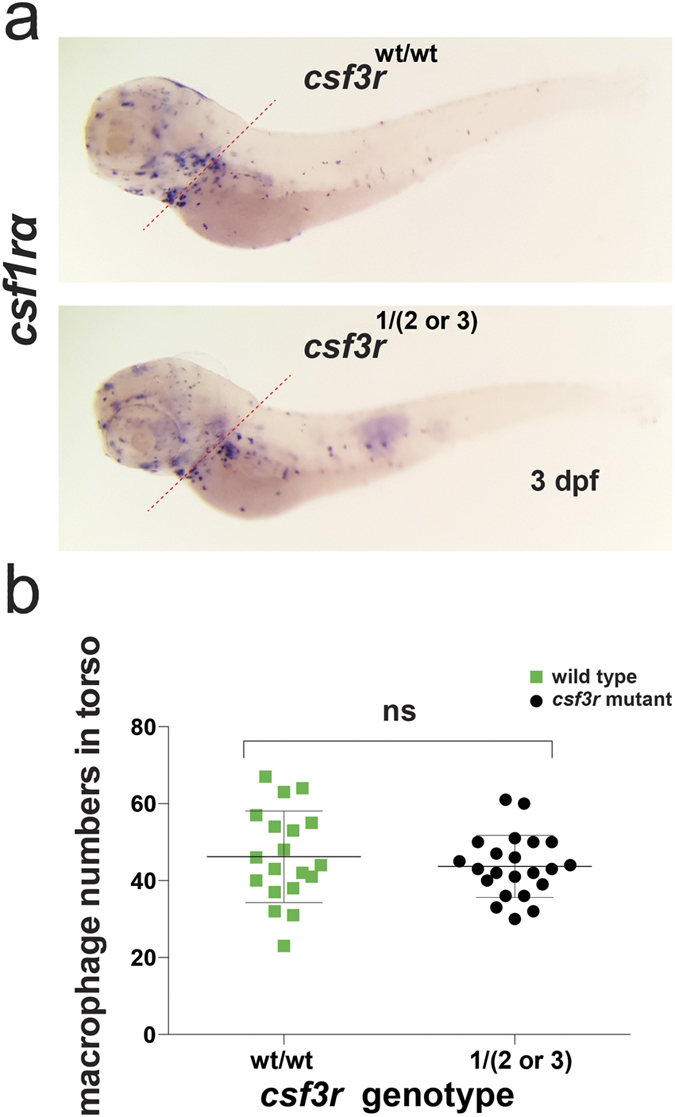
Normal macrophage numbers in *csf3r* null F2 embryos. (**a**) Photomicrographs of representative 3 dpf WT and *csf3r* null embryos stained by whole mount *in situ* hybridisation (WISH) for the macrophage-specific marker *csf1ra/cfms*. (**b**) Quantification of torso-located macrophage numbers in *csf1ra/cfms* WISH embryos in shows no difference between genotypes (the torso being the region distal to the dotted red line in (**a**), selected for scoring for its lower density of macrophages and lower incidence of overlapping cells).

The myeloid deficiency in *csf3r* mutant embryos was neutrophil-specific and did not affect macrophage numbers. To quantify macrophage numbers, whole mount *in situ* hybridisation gene expression analysis for *csfr1a/cfms* was performed. There was no difference between the number of *csfr1a/cfms*-expressing macrophages located in the torso of *csf3r* mutant and WT embryos at 3 dpf (Fig. 3a,b).

### *csf3r* mutant zebrafish are adult viable despite neutrophil depletion in peripheral tissues

**Figure 4:**
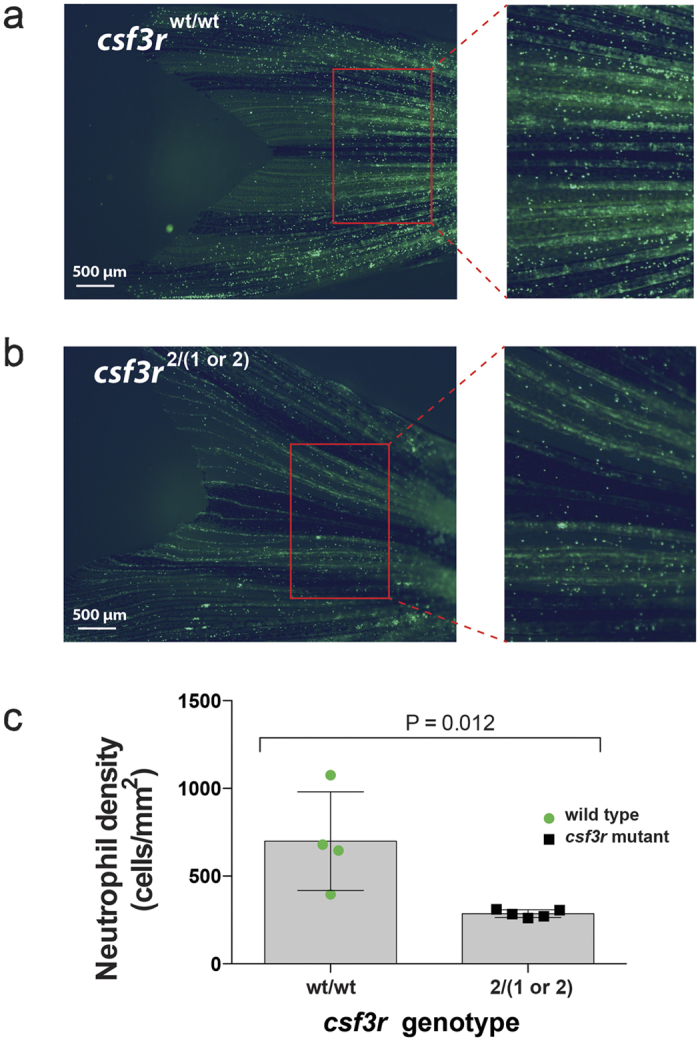
Neutrophil deficiency in *csf3r* null adults. (**a**,**b**) Fluorescent photomicrographs of 5 month old adult *Tg(mpx:EGFP)* zebrafish tail fins showing reduced numbers of EGFP-positive neutrophils in *csf3r* null (**b**) vs WT (**a**) animals. (**c**) Quantification of tail fin neutrophil density. n = 4–5 animals/genotype as shown.

*Csf3r*^*−/−*^ mutant zebrafish were viable as adults of both sexes. This is demonstrated by the six F1 adults incrossed for the analysis of Fig. 2e, which included parent fish with both homozygous and compound heterozygous mutant genotypes (specific allelotypes: *csf3r*^*3/3*^, *csf3r*^*1/2*^, *csf3r*^*2/3*^, and *csf3r*^*1/3*^). These fish retained a neutrophil-depleted phenotype into adulthood (Fig. 4a,b), demonstrated by quantification of neutrophil abundance and density in their tail fins. *Csf3r* mutant animals had a neutrophil density of only 41% of wildtype levels (Fig. 4c).

**Figure 5:**
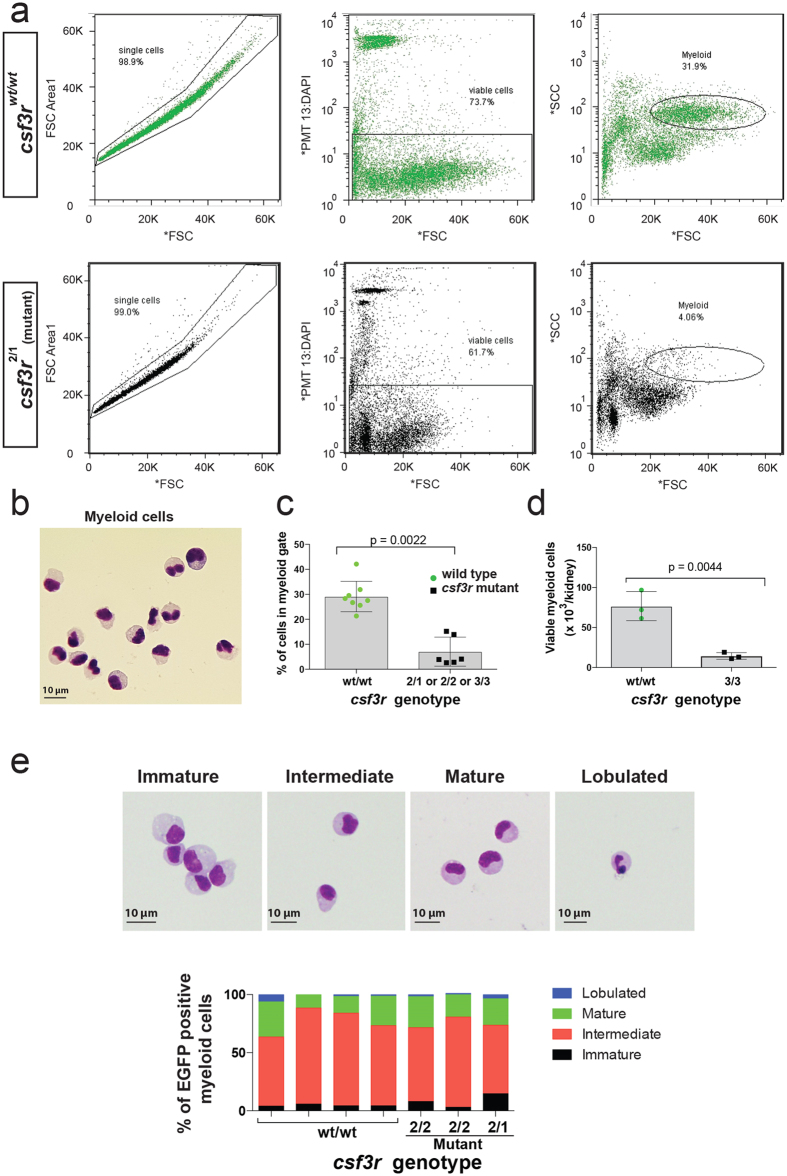
Reduced granulopoiesis in *csf3r* null kidney marrow. (**a**) Representative examples showing gating strategy on single and viable cells, and forward/side scatter profiles (FSC ad SSC) displaying reduced number of cells in the myeloid cell gate of *csf3r*-mutant kidney marrow cells compared to wildtype (WT). Supplementary Fig. S3 provides this information for all samples contributing to data in this figure. (**b**) May Grünwald-Giemsa stained cytospin of WT myeloid gate cells. (**c**) Percentage of viable cells falling within myeloid gate, which is significantly reduced in *csf3r*^*−/−*^ mutant kidney marrows. Points represent different animals. Data are mean ± SD; p-value from Mann-Whitney test. n = 8 animals (WT) and 6 (mutant). (**d**) Number of viable myeloid cells/kidney, which is significantly reduced in *csf3r*^*−/−*^ mutant kidney marrows. Data are mean ± SD; p-value from unpaired, 2-tailed t-test. n = 3 animals/group. (**e**) Four-category neutrophil differential counts based on the categories illustrated (top row of panels) in WT (n = 4) and *csf3r*^*−/−*^ mutant (n = 3) kidney marrow cells, demonstrating no marked difference.

The kidney is the primary haematopoietic and granulopoietic organ in adult zebrafish^8^. Kidney marrow granulopoiesis in adult *csf3r* mutants was assessed by preparing single cell suspensions of adult kidney marrow followed by analysis of population sizes by FACS^12^ and cellular morphology by histological staining. The proportion of kidney marrow cells within the myelomonocytic gate was significantly reduced in *csf3r* mutant kidney marrow compared to wild type (7 ± 6% in *csf3r*^*−/−*^ vs. 29 ± 6% in *csf3r*^*WT/WT*^, p = 0.0022) (Fig. 5a–c). This translated into a 5.3-fold reduction in the absolute number of myeloid cells per kidney (14 ± 4 vs. 77 ± 18 × 10^3^ viable myeloid cells recovered/kidney from *csf3r*^*−/−*^ and *csf3r*^*WT/WT*^ respectively, p = 0.0044) (Fig. 5d).

**Figure 6:**
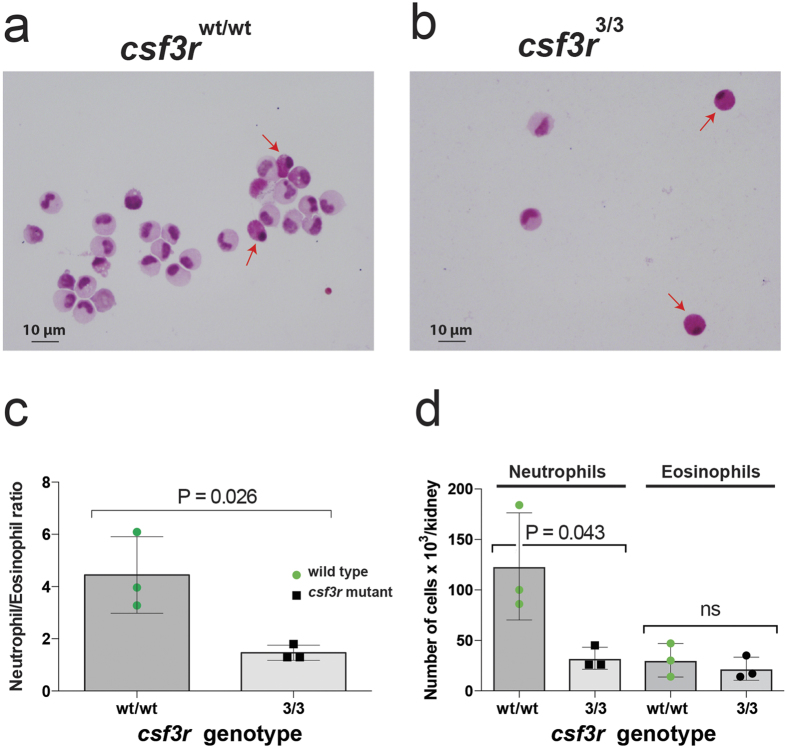
Relative preponderance of eosinophils in *csf3r* null kidney marrow. (**a**,**b**) Periodic Acid-Schiff (PAS) stained cytospins of FACS-purified myeloid cells from WT (**a**) and *csf3r*^*3/3*^ mutant (**b**) kidney marrow. Red arrows indicate eosinophils, recognised by their PAS-positive strongly pink-staining cytoplasm and nuclear morphology and position. (**c**) Higher Neutrophil/Eosinophil ratio in WT vs *csf3r*^*3/3*^ mutant myeloid cells. Individual ratios determined from >150 cell differential counts. (**d**) Although neutrophil numbers are depleted in *csf3r*^*−/−*^ kidney marrow, eosinophil numbers are not, indicating that the change in neutrophil:eosinophil ratio is due to relative change in the cell population sizes.

A 4-category differential count revealed that within the residual population of neutrophil lineage cells, *csf3r*-mutant kidney marrow granulopoiesis showed no marked maturational difference compared to WT (Fig. 5e). The morphologic assessment of myeloid cells in kidney marrow cytospin preparations also revealed an increased proportion of eosinophil granulocytes compared to neutrophil granulocytes (Fig. 6a,b). The neutrophil:eosinophil ratio in Periodic Acid-Schiff stained cytospins prepared from the myeloid gate was 1.5 ± 0.3 in *csf3r*^*−/−*^ mutant vs. 4.4 ± 1.5 in *csf3r*^*WT/WT*^ (p = 0.026) (Fig. 6c). A similar observation was made in May Grünwald-Giemsa stained preparations in an independent experiment (Supplementary Fig. S4). However, this change was relative, because the total numbers of eosinophils in kidney marrow suspensions was not different between wildtype and *csf3r*^*−/−*^ mutants (Fig. 6d).

## Discussion

These new *csf3r*^*−/−*^ mutant zebrafish have a basal neutrophil deficiency that persists into adulthood. This phenotype is concordant with that of *Csf3* and *Csf3r* knockout mice^3^,^4^. The reduced numbers of neutrophils in *csf3r*^*−/−*^ zebrafish indicates that in zebrafish also, there is a requirement for Csf3r signalling to achieve a normal output from granulopoiesis. At the same time, the residual neutrophils present in *csf3r*^*−/−*^ zebrafish indicate that, also in zebrafish, there are Csf3-independent mechanisms still capable of some neutrophil production.

These zebrafish *csf3r*^*−/−*^ mutants also provide a new animal model of the rare form of autosomal recessive human severe congenital neutropenia due to biallelic *CSF3R* mutations (SCN7, OMIM #617014)^5^,^6^. The five mutant *CSF3R* alleles in the five patients described with SCN7 were nonsense mutations in either the extracellular cytokine homology domain (n = 1 allele), fibronectin-like domains (n = 3 alleles) or a p.Arg308Cys missense mutation in the cytokine homology domain (n = 1 allele)^5^,^6^. The more N-terminal *csf3r* mutations in this zebrafish allelic series occur in the immunoglobulin domain and best model the nonsense human SCN disease alleles. Furthermore, several of the human patients described were compound heterozygotes carrying two different *CSF3R* mutations^5^,^6^, a similar scenario to the compound heterozygotes resulting from our CRISPR/Cas9 mutagenesis and breeding strategy. The N-terminal nonsense mutations in the zebrafish mutants would not be expected to carry the leukaemogenic risk associated with the various C-terminal truncations that are prevalent as acquired somatic mutations in long-standing severe congenital neutropenia patients and some acute myeloid leukaemias, and which transmit hyperproliferative signals^7^. Interesting, the marrow of *csf3r*^*−/−*^ mutant zebrafish had a proportional over-representation of eosinophils, a feature observed in some human cases of severe congenital neutropenia^13^,^14^.

Despite the lack of concordance reported between some zebrafish mutant phenotypes and that of their corresponding morphants^15^,^16^, for *csf3r* mutants and morphants, this aspect of the phenotype is concordant and confirms an absolute requirement for *csf3r* signalling in embryonic zebrafish granulopoiesis for which there are no compensatory genetic mechanisms.

We did not observe a reduction in macrophage numbers in *csf3r*^*−/−*^ mutant embryos at 72 hpf, although at this time point, embryos with *csf3a* and *csf3b* overexpression from mRNA injections had increased macrophage numbers^10^, suggesting there are *csf3-*dependent signalling pathways in zebrafish that when driven strongly, are capable of impacting on macrophage abundance. In *csf3r* morphants, at 22 hpf macrophage numbers were normal, whereas at 96 hpf a ∼10% reduction was observed^2^. In both our mutants and these morphants, macrophages were scored as cells expressing *csf1ra/cfms* by whole mount *in situ* hybridisation. Whether or not there is a macrophage deficiency in older *csf3r*^*−/−*^ mutant embryos or adults awaits further comprehensive studies; we suggest that this is best assessed by a range of macrophage markers such as crossing the mutant alleles onto *mpeg1-* or *mfap4-*driven fluorophore reporter lines^17^,^18^, which will take several generations.

There has been a considerable need in the field for methods to assess the specific contribution of neutrophils in zebrafish models of developmental processes, and in disease/biomedical models such as inflammation, infection and regeneration^19^. These neutrophil-depleted *csf3r*^*−/−*^ mutant animals provide a new tool for doing this, with the advantages of specificity, reproducibility and persistence of the neutrophil-deficiency phenotype.

In particular, for studies that seek to assess the requirement or contribution of neutrophils to biological processes or disease models, this series of *csf3r*^*−/−*^ mutants offers significant advantages over transient morphant knockdown approaches. These include lack of potential toxicity and no requirement for external manipulation, genetic stability, phenotype reproducibility and persistence, and concordance between different mutant allelotypes. There are 4 different *csf3r* morpholinos used in studies reported to date, targeting different regions of the Csf3r protein^2^,^17^,^20^,^21^. Although the *csf3r* morphant neutrophil deficiency phenotype is generally concordant for these different morphants, a direct comparison of all these reagents has not been conducted and the possibility remains of there being at least subtle differences between them in some or all of phenotype strength, persistence, and also potentially in confounding non-documented off-target effects. Groups of morphants also have heterogeneity reflecting the unavoidable variability inherent in dosing and delivery of morpholino oligonucleotides.

In other zebrafish mutants that have neutrophil deficiency, it occurs in the context of confounding multisystem phenotypes and often with accompanying macrophage deficiency. These mutants include, among many others: *cloche/npas4*^22^,^23^, *alk8*^24^, *med12*^25^, *prpf8*^26^, *plcg1*^27^ and *runx1*^28^.

Experimentally, other transient genetic approaches have been used to deplete embryos of leukocytes including neutrophils. Normal lineage-balanced myelopoiesis requires appropriate levels of *pu.1/spi1b*, and *pu.1/spi1b* morphants have been used to induce a leukocyte-depleted state. However, the *pu.1/spi1b* morphant lacks specificity for neutrophil depletion and the outcome is highly dose-dependent: with modestly lowered Pu.1/Spi1b levels only macrophage development is impaired, and it is only with very substantive Pu.1/Spi1b depletion that neutrophil development is also impaired^28^,^29^. Imbalance of *irf8* signalling also perturbs the balance of neutrophil/macrophage production in zebrafish embryos, and has often been used to do so, but in this case, the suppression of neutrophil production achieved by *irf8* overexpression is accompanied by increased macrophage numbers, and so lacks specificity of effect^30^,^31^.

Although the genetically stable neutrophil deficiency of *csf3r*^*−/−*^ mutants that persists through embryonic, larval and adult life is an advantage for some experimental questions, it also has its drawbacks. It provides a tool enabling the requirement for normal basal granulopoiesis to be evaluated in experimental models, but it complicates the assessment of emergency granulopoietic responses, including those driven by other haemopoietins, which in *csf3r*^*−/−*^ mutants would be initiated from an already depleted basal state. It also does not permit the evaluation of the impact of acute, sudden neutrophil depletion on experimental models. A conditional system for achieving acute neutrophil ablation is provided by using our *Tg(mpx:kalTA4)*^*gl28Tg*^ transgenic line to drive neutrophil-specific expression of the E. *coli nitroreductase (NfsB*) protein^32^. This approach has been employed in embryos (achieving up to 95% neutrophil depletion from 4 days of induction), but not yet in adults^32^.

In summary, we have built a new animal model of severe congenital neutropenia due to CSF3R mutation. These *csf3r*^*−/−*^ mutant zebrafish will also be an extremely valuable resource for assessing the contribution of neutrophils in zebrafish developmental and disease models.

## Methods

### Zebrafish, husbandry and animal ethics

Fish were held in FishCore (Monash University) using standard practices. Embryos were incubated in egg water (0.06 g/L salt (Red Sea, Sydney, Australia)) or E3 medium (5 mM NaCl, 0.17 mM KCl, 0.33 mM CaCl_2_, 0.33 mM MgSO_4_, equilibrated to pH 7.0). From 12 hpf, 0.003% (w/v) 1-phenyl-2-thiourea (Sigma-Aldrich) was used to inhibit pigmentation. Embryos were held at 28 °C in an incubator (Thermoline Scientific) following collection. The *Tg(mpx:EGFP)*^*il114Tg*^ strain^33^ was used, which has been maintained on a majority Tübingen background by outcrossing to the Tübingen strain at least every second generation 1–2/yr for >10 years. The four new *csf3r* alleles called c*sf3r*^1^,^2^,^3^,^4^ in this report (Fig. 1a) have been designated *csf3r*^*gl*^^31^,^32^,^33^,^34^31*–*34 respectively on the Zebrafish Information Network (ZFIN)^34^. Animal experiments followed National Health and Medical Research Council (NHMRC) guidelines (“Australian code of the care and use of animals for scientific purposes” 8th edition, NHMRC, 2013), were approved by the Monash University Animal Ethics Committees (protocol MAS-2010-18) and were conducted in accordance with these protocols.

### CRISPR/Cas9 mutagenesis of zebrafish *csf3r*

CRISPR/Cas9 mutagenesis was based on the method of Gagnon *et al*.^35^. Briefly, the web tool “CHOPCHOP” was used to design a set of three sgRNA molecules (designated C1 to C3) to target the zebrafish *csf3r* gene. All three sgRNAs targeted exon 2. Each sgRNA contained a gene-specific spacer sequence (sequences in Fig. S1c) followed by a Cas9 enzyme binding sequence. These two sites were initially provided in different oligonucleotides called “site specific” and “constant” respectively. Oligonucleotides (Sigma) were annealed and single-stranded overhangs were filled in by T4 DNA polymerase (NEB) activity to form a double-stranded oligonucleotide, dsDNA (120 nucleotides after annealing and extension). All steps were performed in a 96-well T100™ Thermal Cycler (Bio-Rad). This was used as a template for *in vitro* transcription of sgRNA using mMESSAGE mMACHINE^®^ T7 Transcription Kit (Ambion) followed by RNA clean up using Sephadex G-50 spin columns (Roche Diagnostics) according to the manufacturer’s instructions. synthesised sgRNA integrity was checked on a non-denaturing 1% TBE gel. For microinjection, individual sgRNAs (50–200 ng/μL) were mixed with Cas9 Nuclease 20 μM (New England Biolabs) at a 1:1 ratio and microinjected (500–1000 pg) directly into 1-cell *Tg(mpx:EGFP)* embryos.

### Genotyping

DNA was extracted from single embryos or fin clips from adult fish using the HotSHOT protocol^36^ and purified PCR or gel extracted PCR products (AccuPrep^®^ PCR/Gel purification kit, BIONEER) were sent for sequencing. 25 μL PCR reactions consisted of 0.5 μL Phusion High Fidelity DNA Polymerase (Thermo scientific), 5 μL 5X Phusion HF Buffer, 2 μL dNTP (2.5 mM), 0.5 μL forward primer (10 μM) (5′CCTTGCACATTTACTACCGACA3′), 0.5 μL reverse primer (10 μM) (5′GTCCTCCTGAACACACACAAGA3′), 5 μL genomic DNA and 11.5 μL of nuclease free water. Biorad T100 thermal cycler with the following program was used for amplification: 90 seconds at 95 °C as initial denaturation followed by 30 cycles of 30 sec at 95 °C for denaturation, 30 sec at 66 °C for annealing, 30 sec at 72 °C for extension, and final extension at 72 °C for 5 min. Sequencing was performed using the reverse primer (Micromon sequencing facility, Monash University). Sequencing traces were analysed using DNASTAR (Version 14) and ApE (A Plasmid Editor v.2.0.47). Mutations were identified manually by comparing mutant and wild-type traces.

### Microscopy and neutrophil enumeration

An Olympus MVX10 microscope fitted with an Olympus DP72 camera and CellSens software version 1.11 was used for fluorescence and bright field imaging. EGFP-expressing neutrophil granulocyte numbers were quantified at various time points in the tail region caudal to the yolk extension, which includes the leukocyte-rich caudal haematopoietic tissue (CHT). Manual counting of neutrophil numbers was assisted by the brush tool in Paintbrush 2.1.2 (Soggy Waffles), which records clicks to avoid duplicate counting. For Supplementary Fig. S2 data, “Neutrophil Units” refer to a neutrophil number determined with assistance of the “Find maxima” function in Fiji (Version 1.47n).

Neutrophil density in adult tail fins was determined from *Tg(mpx-EGFP)* tail fin images of the appropriate *csf3r* genotype anaesthetised with 160 mg/L tricaine.

### Whole-mount *in situ* hybridisation

Whole-mount *in situ* hybridisation was performed as previously described^22^,^37^ using a *csf1ra* digoxigenin-labelled riboprobe^38^. Cells were counted assisted by the Paintbrush 2.1.2 (Soggy Waffles), which records clicks to avoid duplicate counting, and by zooming to optimally resolve aggregates of cells into single cells.

### Adult neutrophil isolation and evaluation

Kidneys were dissected from adult zebrafish euthanased by dense anaesthesia with 300 mg/L tricaine. A kidney single cell suspension was prepared for FACS in ice-cold 0.9X PBS containing 5% FBS^12^. Yield of viable cells per kidney was determined by trypan dye exclusion using a haemocytometer. FACS analysis was performed in Monash Flowcore using an Influx2 cell sorter (BD Biosciences) and data were analysed using FlowJo software (version 7.6.1). Gating strategies for all samples are displayed in Supplementary Fig. S3. For sorting cells to generate the histological images and differential counts of Fig. 5b and e, the myeloid gate shown was carefully reproduced by eye for each sorted sample. Cytospins of FACS-purified myeloid cell populations (gated as displayed in Fig.5a) were prepared at 800 r.p.m. for 5 minutes using a Shandon Elliott Cytospin machine and stained with May Grünwald-Giemsa^22^. Periodic Acid-Schiff staining used undiluted May Grünwald solution (VWR Biosciences 352622 M) as fixative followed by staining with Periodic Acid-Schiff according to the manufacturer’s instructions (Sigma 395B-1KT).

### Statistics

Descriptive and analytical statistics were generated in Prism 6.0 f (GraphPad Software). Throughout this report, parametric data are presented as mean ± SD. n values are indicated by dots in histograms; every individual n value represents a different animal (i.e. there was no replicate scoring within a single animal). Statistical analysis used unpaired two-tailed t-tests, Mann-Whitney test, one-way ANOVA with Tukey’s multiple comparisons test, and the Wilcoxon rank sum test. p < 0.05 was taken to indicate a significant difference.

## Additional Information

**How to cite this article:** Pazhakh, V. *et al*. A GCSFR / CSF3R zebrafish mutant models the persistent basal neutrophil deficiency of severe congenital neutropenia. *Sci. Rep.* **7**, 44455; doi: 10.1038/srep44455 (2017).

**Publisher's note:** Springer Nature remains neutral with regard to jurisdictional claims in published maps and institutional affiliations.

## Supplementary Material

Supplementary Figures
