# Supplementary material for: A GCSFR/CSF3R zebrafish mutant models the persistent basal neutrophil deficiency of severe congenital neutropenia
Source: Sci Rep. 2017 Mar 10;7:44455. doi: 10.1038/srep44455 (PMC5345067; doi:10.1038/srep44455)

A GCSFR / CSF3R zebrafish mutant models the persistent basal neutrophil deficiency of severe congenital neutropenia

Vahid Pazhakh, Sharon Clark, M. Cristina Keightley, Graham J. Lieschke

SUPPLEMENTARY FIGURE LEGENDS

Figure S1. Targeting strategy for CRISPR/Cas9 mutagenesis of zebrafish c*sf3r* locus (supports Figure 1).

a. Target sites of C1, C2 and C3 sgRNAs, mapped to zebrafish csf3r cDNA (transcript ID = csf3r-201 ENSDART00000063986). Respective target sites in exon 2 are colour-coded. C1 and C3 targeted the (+) strand, whereas C2 targeted the (-) strand.

b. Sequencing chromatograms annotated to show the four different mutant *csf3r* alleles in two homozygous and two compound heterozygous configurations. PAM=protospacer adjacent motif. Blue shading indicates sgRNA target site.

Figure S2. CRISPR/Cas9 mutagenesis of zebrafish *csf3r* locus in F0 embryos (supports Figures 1 and 2).

a. WT sequence trace in control uninjected embryos.

b. Sequence traces from four different F0 C3 sgRNA-injected embryos with mosaic *csf3r* genotypes commencing in the vicinity of the sgRNA target site (blue shading). PAM=protospacer adjacent motif.

c. Neutrophil abundance, represented by Neutrophil Units in the tail, of 3 days post-fertilization (dpf) F0 embryos injected with three different sgRNAs (C1, C2, C3), compared to control uninjected embryos. Data are mean±SD. p-values from unpaired t-test, ns=not significant. Only sgRNA C3 lowered neutrophil abundance in F0 embryos.

Figure S3. FACS gating strategy for determining neutrophil abundance in adult kidney marrow single cell suspensions (supports Figure 5).

FACS gates and profiles for n=8 WT and n=6 *csf3r*$^{-/-}$ adult kidney marrow single cells suspensions contributing to the morphological and quantitative analyses in Fig. 2b-e.

Figure S4. Relative preponderance of eosinophils in *csf3r* null kidney marrow (supports Figure 6).

a-b. May Grünwald-Giemsa stained cytospins of FACS-purified myeloid cells from WT and *csf3r*$^{1/2}$ mutant kidney marrow. Red arrows indicate eosinophils, recognised by their characteristic granular cytoplasm and nuclear morphology and position. Insets near corresponding eosinophils highlight at higher magnification their characteristic cytoplasmic and nuclear morphology that distinguishes them from the adjacent myeloid cells (eosinophils in dashed red circles).

c. Higher Neutrophil/Eosinophil ratio in WT vs c*sf3r*$^{1/2}$ mutant myeloid cells. Individual ratios determined from >200 cell differential counts.

Supplementary figure S1

a sgRNA target sites map

Exon-1

ATGGCATCCGTCTCGCTTGTGCTTCTGCTGTGGATGTATATTTATGTTGTGATAAAAG

gtaaatatacatttttacaaa.......1,947 nt.......ttgcacatttactaccg

Exon-2

TGACTGGAGCATCATCGTGTGGGAATGTCTACACGCCTGCGCCT**GTGGTTTTGGCTGGAT** C3
CTCCGGTTTCTGTGTCCTGCTCCATCGAGGAAGACTGCCCCCTGACCAAAG**GCAAAGTGT** C2
TTTACGTGCAGTGGAGGATCGACGGCCAGGTTGTCCCAAGAAATTACACCAATCAGGAGA
GTAACATGACATACAGTGTGCTCATA**CCTCGCCTTCAAGACACAAGCGC**AAGCATACTCT C1
GTCTTGTGTGTGTTCAGGAGGACTGCCAGATTGTGGATGGAGTGGAAGTTAAAAATGGAA

gtgagtatgtgcttgaattcatatc.......2,128 nt.......aaatattcagatt
ttgtctttacag

Exon-3

ATCCGCCAGTTCCTCATAACCTGAGCTGTGCTTTGACTTTAGTGAAAATGCCCAGCCTCC
GGTGTGACTGGAATCCTGTTCAGGAAATAGCAAATCTTCCTACCAACTATACTCTGCATT
TATTTAG

b

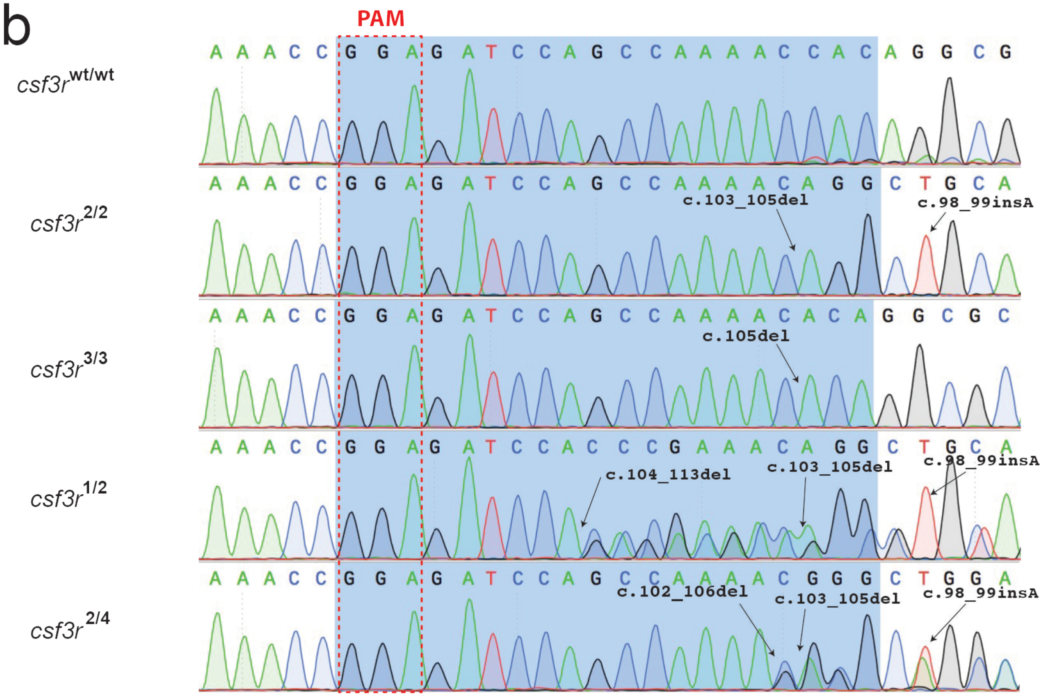

Supplementary figure S2

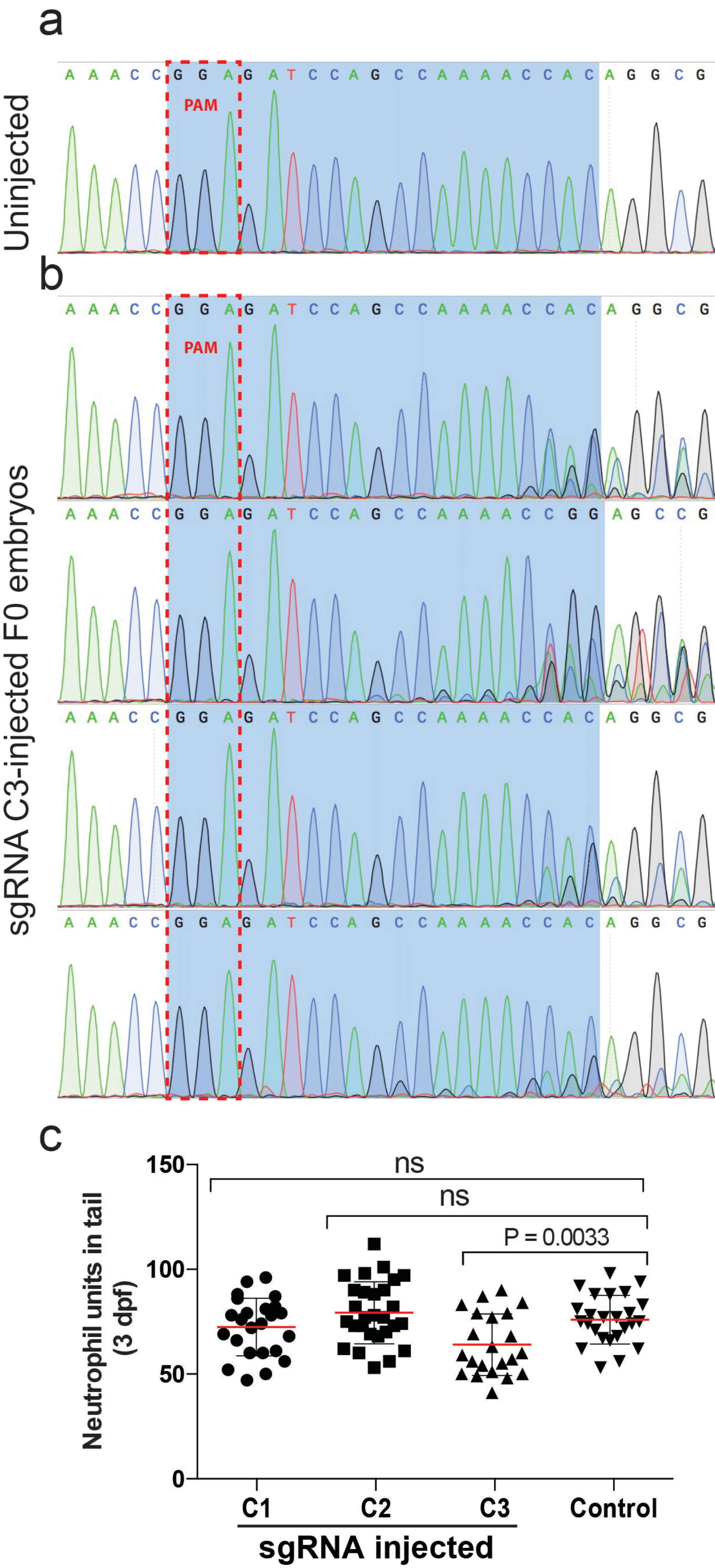

Supplementary figure S3

Myeloid cell gate in adult fish kidney marrow

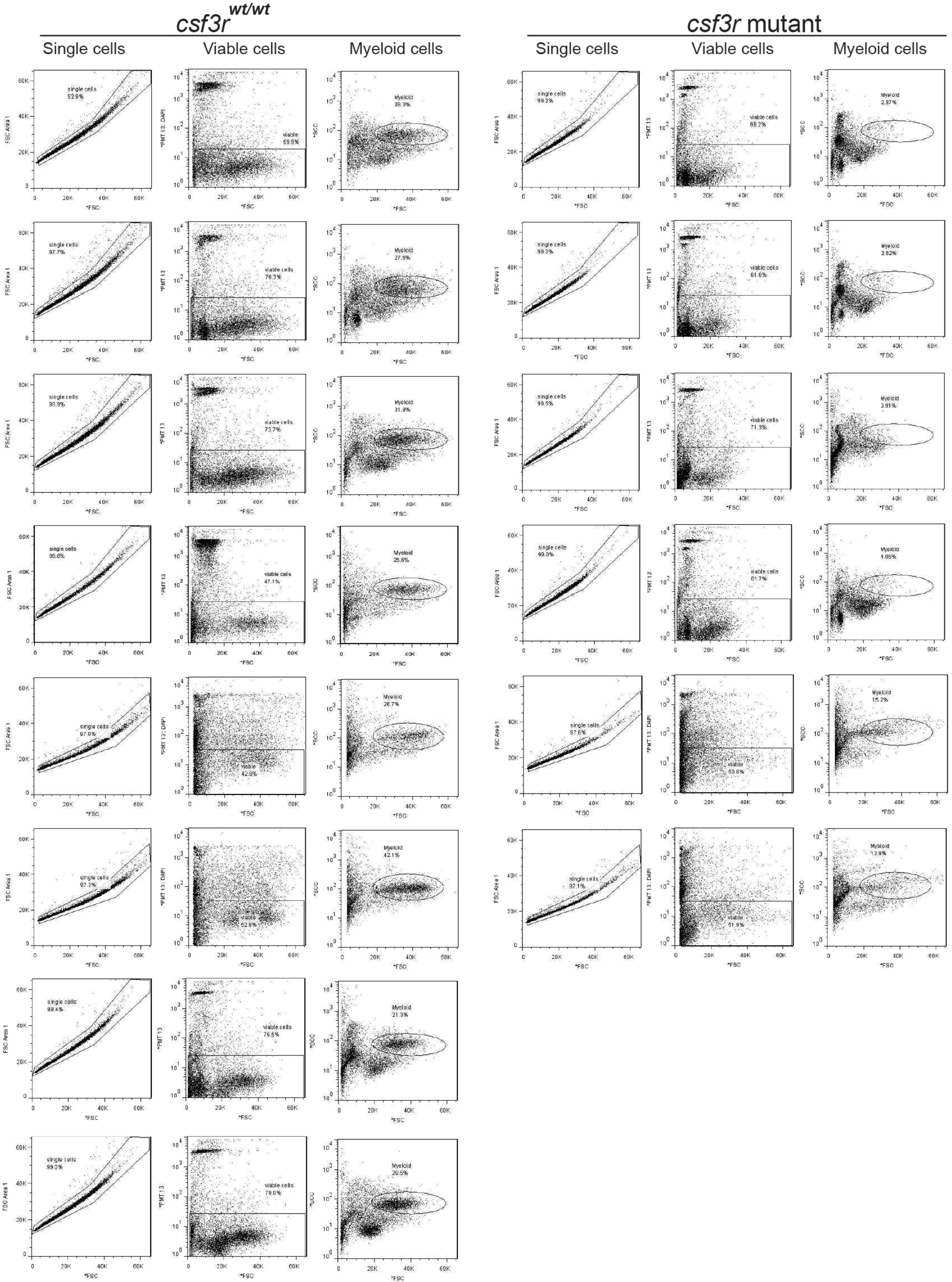

Supplementary figure S4

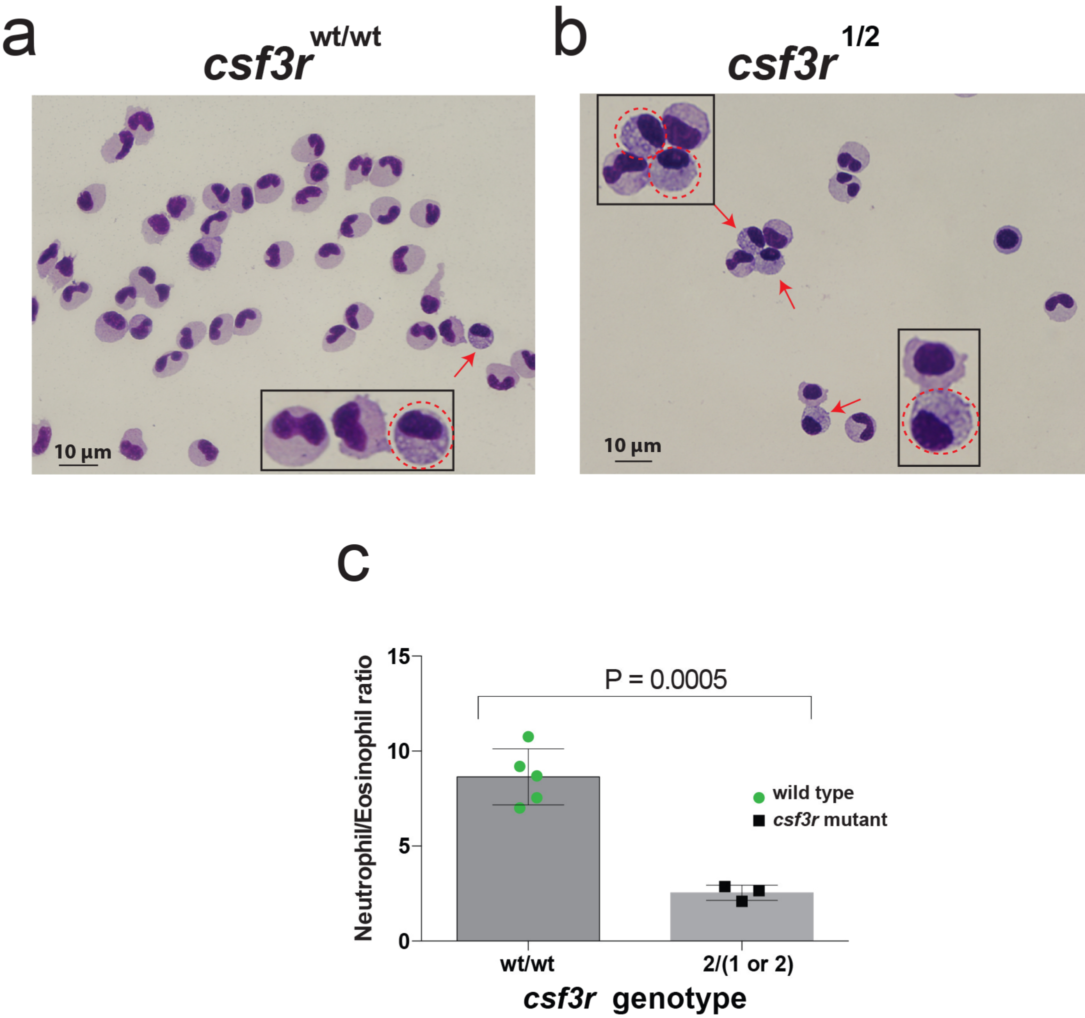

Supplement: Supplementary Figures [file srep44455-s1.pdf]
